# Epigenetic Influences on Wound Healing and Hypertrophic-Keloid Scarring: A Review for Basic Scientists and Clinicians

**DOI:** 10.7759/cureus.23503

**Published:** 2022-03-26

**Authors:** Asia Thomas, Kanith Farah, Richard M Millis

**Affiliations:** 1 Pathophysiology, American University of Antigua, Coolidge, ATG

**Keywords:** collagen cross-linking, oxidative stress, human genetics and epigenetics, keloid scar, hypertrophic scar

## Abstract

Primary care physicians and dermatologists are challenged by patients affected by keloid or hypertrophic scarring resulting from accidental wounding, surgical incisions, tattooing, or “branding” procedures to demonstrate their association with a specific culture, fraternity, or cult. The dysregulated wound healing associated with keloids and hypertrophic scarring adversely affects genetically susceptible individuals, especially persons of color with Fitzpatrick Skin types IV-VI. Although the specific mechanisms of bulky hypertrophic/keloid scarring and its association with oxidative stress and inflammation remain unclear, the current knowledge base is sufficient to provide some guidance to health practitioners who must serve, treat, and counsel affected individuals. This review focuses on providing insight to healthcare professionals about the role of epigenetics, oxidative stress, poor local oxygenation, and its relationship to impaired wound healing. The goal is to promote further research on bulky hypertrophic and keloid scarring for its prevention and to develop evidence-based clinical guidelines for optimal treatment.

## Introduction and background

Keloid and hypertrophic scarring are found equally in both males and females, in persons 20-30 years of age [1] and in those with Fitzpatrick skin types IV-VI [2,3]. Bulky hypertrophic and keloid scarring is, largely, a manifestation of oxidative stress, and the role of reactive oxygen species (ROS) in dysregulated wound healing is, therefore, a topic of considerable interest. The science of epigenetics differentiates diseases using information about how a person’s external environment influences their genes, primarily the expression of the gene products or proteins, without changing their DNA. This review informs health practitioners about epigenetic influences on wound healing which may be applied to improving treatment strategies for persons affected by bulky and keloid scarring.

Bulky scarring appears to be a result of dysregulated wound healing. Wound healing occurs in three phases: inflammation, proliferation, and remodeling. Excessive inflammation or poor local oxygenation causes distorted wound repair and produces excessive amounts of free radicals and ROS. Tissue mediators such as transcription growth factor-beta (TGF-beta) induce angiogenesis and generate wound fibrosis [4,5]. When overexpressed, TGF-beta stimulates connective tissue growth factor (CTGF) which ultimately forms hypertrophic and keloid scarring [6].

## Review

Epigenetics, genes, and histones

**Figure 1:**
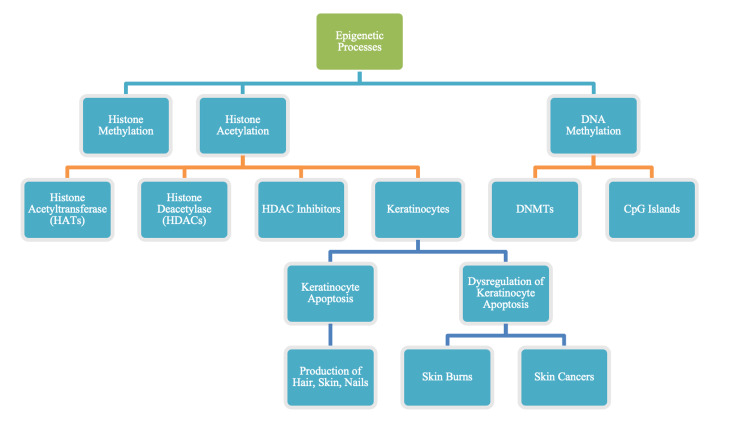
Histone methylation, histone acetylation and DNA methylation are integral epigenetic processes that keep phenotypes healthy. Dysregulation of epigenetic processes can alter gene expression, causing apoptosis and skin cancers. Histone acetylation is essential for the production of keratinocytes and subsequently leads to the formation of hair, skin, and nails. DNA methylation also plays a role in this epigenetic regulation at a different level. Histone acetylation only plays a role when transcription is initiated by the binding of the transcription complex to the promoters of genes DNMTs: DNA methyltransferases; CpG Islands: A stretch of DNA with a high quantity of the nucleotides G and C next to one another

Keloid-causing genetic variants have been identified in African-American, Japanese and Chinese families [7]. Changes in the genetic expression of certain proteins may alter a person’s physical and physiological characteristics, known as phenotype. Fitzpatrick skin types are a good example of interindividual variations in phenotype. Three highly structured epigenetic processes have evolved to protect genes from random gene expression and to maintain healthy skin phenotypes: namely, (i) histone methylation, (ii) histone acetylation, and (iii) DNA methylation. The regulation of histone modifications is via the activity of histone acetyltransferases (HATs) and histone deacetylases (HDACs). The mechanism of acetylation by HATs involves the transfer of negatively charged acetic acid groups from acetyl-CoA to the amino-terminal tail of the lysine residues in the amino tail of the core histone proteins, which neutralizes the histone and dissociates it from the DNA. Acetylation exposes the DNA and increases the transcriptional potential of a targeted gene [8]. HDACs catalyze the removal of acetyl groups of histones, thus causing a switch back to the histone’s natural cationic form. This allows binding to the DNA, thereby decreasing the transcriptional potential of the targeted gene [8]. HDAC inhibitors are used to inhibit the growth of benign neoplasms and cancerous tumors by promoting the expression of p21 which inhibits the cell cycle and promotes cell differentiation [9]. Keratinocytes, the main cell type in the human epidermis, undergo a unique form of apoptosis that leads to cornification involving skin, hair, and nails. Dysregulation of keratinocyte apoptosis is an important factor in sunburn and skin cancers [10]. Figure 1 summarizes the epigenetic processes relevant to cutaneous wounding and other affectations of the skin.

DNA methylation

Both histone and DNA methylation are epigenetic processes that regulate gene expression and ensure that genes are expressed in a healthy and structured manner; dysregulation may lead to various abnormal conditions, including keloids and hypertrophic scarring [11]. DNA methylation is a tightly regulated process that plays a key role in embryogenesis and environment-gene interaction throughout life. DNA methylation is catalyzed by a family of DNA methyltransferases (DNMTs) which transfer a methyl group from S-adenyl methionine (SAM) to the fifth carbon of a cytosine residue to form 5-methyl-p-cytosine [12,13]. A large proportion, although not all, of human genes, initiate transcription from promoter regions of the genome which have an elevated content of CpG dinucleotides and G+C base pairs known as CpG islands. Within the promoter, CpG islands are free from DNA methylation only when they are being expressed. Silenced genes have highly methylated CpG islands. This is purported to account for a lower mutation rate within promoter regions of such genes. DNA methylation results in the repression of gene transcription and appears to be a critical regulator of aging and genomic imprinting. Expression of DNMT1 in hair follicles and basal epidermis is decreased by cell differentiation [13]. Experimental ablation of DNMT1 in mice causes hyperplasia of sebaceous glands, thickened epidermis, and upregulation of various cell differentiation biomarkers [13]. These findings reflect the classical role of DNA methylation as a suppressor of gene transcription and, therefore, gene expression. DNA methylation is also an important component of oncogene expression and the development of cancer. Transcriptional silencing and gene inactivation are associated with hypermethylation. In contrast, hypomethylation is linked to chromosomal instability and loss of imprinting characterized by the inability to transfer methylation patterns to daughter cells [14].

**Figure 2:**
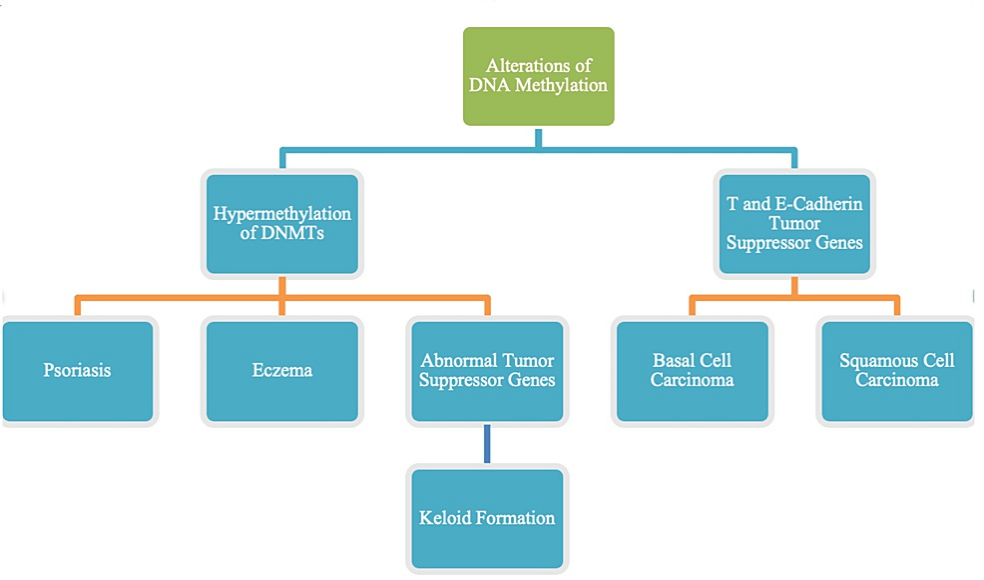
The role of DNA methylation in keloids. DNA methylation is responsible for suppressing gene transcription and expression. Expression of oncogenes promotes both carcinogenesis and keloid formation. DNMTs: DNA methyltransferases

Upregulation of DNMTs causes hypermethylation of some tumor suppressor genes and downregulation of DNMTs causes hypomethylation of some oncogenes [14]. Indeed, DNA methylation is reported to play a critical role in carcinogenesis, especially in the skin. For example, basal and squamous cell carcinomas are reported to result from hypermethylation of T- and E-cadherin tumor suppressor genes [13]. Alterations in DNA methylation may also be causes of certain skin disorders, such as eczema, psoriasis, and seborrheic dermatitis. For example, psoriasis is shown to be associated with DNA hypermethylation which disinhibits a p16 endogenous cell cycle inhibitor that permits normal progression and timing of the first growth phase known as G1 (growth1). Hypermethylation downregulates p16INK4A and inhibits the phosphorylation of retinoblastoma tumor suppressor protein downstream of p16INK4A. The end result of such hypermethylation is abnormal, too rapid progression of cells from cell cycle phase G1 to S (DNA synthesis), characteristic of many cancers [13]. The equilibria associated with DNA methylation are complex and incompletely understood; the aforementioned explanations are overly simplistic and await future research. Whereas hypermethylation can cause downregulation and inhibit transcription and synthesis of tumor suppressor proteins, hypomethylation can destabilize the DNA and be a precursor for oncogenesis. The significance of oncogenesis in the development of keloids is underscored by findings that aberrant methylation of tumor suppressor genes appears to be a factor in the development of keloids [15] and patients affected by keloids exhibit increased incidences of various skin cancers [16]. Figure 2 summarizes the role of DNA methylation in the formation of keloids.

Base-excision repair and DNA methylation

DNA glycosylases are unique enzymes in that they protect the DNA against mutagenesis by virtue of their high specificity for distinct lesions. Upon binding to the lesion, the glycosylase cleaves the N-glycosidic bond, separating the damaged base from its deoxyribose sugar moiety, creating an apurinic/apyrimidinic (AP site) [17]. AP sites are DNA locations where spontaneous hydrolysis can occur, thereby increasing mutation rates when replicated [17]. AP-endonuclease removes nucleotides at the 5′ end of the deoxyribose phosphate, causing a single-strand break or nick in the DNA [17]. Lyase cleaves the 3′ end of the sugar backbone and DNA polymerase beta fills the opening, finally, DNA ligase seals the lesion. Base-excision repair is shown to ameliorate oxidative damage to the DNA in the ultraviolet(UV)B-exposed fibroblasts from the skin of patients with xeroderma pigmentosa [18]. Similar oxidative damage to DNA is likely to occur in the skin of patients with keloids [19]; however, the role of base-excision repair and its relationship to DNA methylation has not been studied in keloids.

Hexose monophosphate pathway shunt and its relation to the respiratory burst

ROS and nicotinamide adenine dinucleotide phosphate hydrogen (NADPH) produced by the hexose monophosphate pathway (HMP) shunt, also known as the pentose phosphate pathway (PPP), play important, complex roles in both the inflammation and aberrant wound healing associated with keloids. NADPH is a vitamin-based cofactor necessary for many reductive reactions. Nicotinic acid and nicotinamide, collectively referred to as niacin, are nutritional precursors of the bioactive molecules nicotinamide adenine dinucleotide (NAD) and nicotinamide adenine dinucleotide phosphate (NADP) [20]. NAD and NADP+ are important cofactors for most cellular redox reactions, and as such are essential to maintain cellular metabolism and respiration [20]. To generate NADPH, the reducing power stored in the bonds of organic molecules is used to reduce NADP+ to NADPH [21]. NADPH is a key component of cellular antioxidant systems [22] and NADPH-assisted reduction of glutathione is utilized in neutrophils, red blood cells, and synthesis of fatty acids for detoxification of peroxides and free radicals. If this requirement is not met, oxidative stress occurs, characterized by exaggerated inflammation [23]. ROS play a pivotal role in the orchestration of the normal wound-healing response [23] by functioning as secondary messengers to many immunocytes and non-lymphoid cells, involved in the repair process. ROS appear to be important in coordinating the recruitment of lymphoid cells to the wound site for effective tissue repair [23].

**Figure 3:**
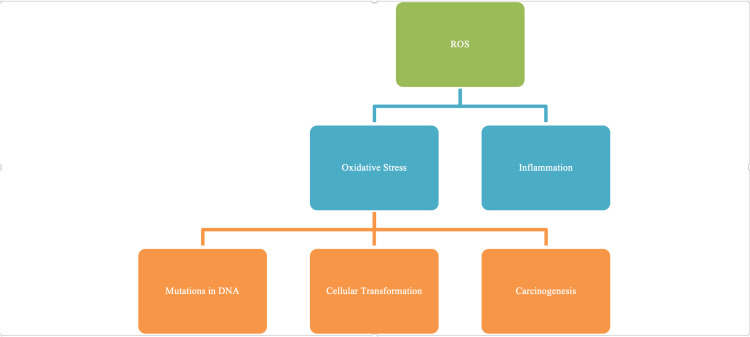
Reactive oxygen species (ROS) and the natural response to wound healing and repair. Oxidative stress alters gene expression and leads to certain cancers and the formation of keloids.

During the inflammatory phase of wound healing, neutrophils and macrophages invade the wound. Neutrophils act as first responders to cellular defense, arriving within minutes to a wound site and regulating the ensuing repair mechanisms after tissue damage [24]. Subsequently, lymphocytes and monocytes also invade the wound tissue and differentiate into activated macrophages. Neutrophils and macrophages produce large amounts of superoxide radical anions and other reactive oxygen species such as peroxides and hydroxyl anions, a phenomenon that is often described as the “respiratory burst.” Fibroblasts can also be stimulated by pro-inflammatory cytokines to produce additional ROS [25]. The respiratory burst has a phagocytic function in which the phagosomes of neutrophils acquire the capacity to eradicate microorganisms. Oxygen is used as a substrate in the phagosome membrane’s NADPH oxidase complex. The role of NADPH can be compared to a light switch wherein, depending on the NADPH/NADP+ ratio, ROS production can be turned on or off; decreased NADP+/NADPH favors ROS production. Superoxide dismutase is another key component of the oxidative burst. Superoxide radical anions (O^2-^) produce the two molecules oxygen and hydrogen peroxide by superoxide dismutase [25]. Hydrogen peroxide and other ROS inhibit the migration and proliferation of various cell types, including keratinocytes [25]. Inhibition of migration and proliferation of keratinocytes adversely affect wound epithelialization and, ultimately, wound healing. ROS is associated with increased expression of the GLUT1 glucose transporter in the skin of patients affected by diabetes [26], psoriasis [27], and keloids [16], thereby providing a biochemical marker for oxidative stress and the potential for developing fibroproliferative lesions. Increased ROS in fibroblasts from keloid scar tissue exposed to hypoxia [28] also suggests that oxidative stress likely plays an important role in the development and maintenance of keloids. Figure 3 demonstrates the connection between ROS and alterations in DNA and fibroblasts for hypertrophic-keloid scarring and carcinogenesis.

Various medical conditions impair wound healing

In addition to malnutrition, there are several lifestyle and medical conditions which delay wound healing including, but are not limited to, obesity, cigarette smoking or other nicotine usages, autoimmune conditions, and comorbidities such as hypertension and diabetes. In diabetic wounds, for example, various dysregulated cellular functions include defective chemotaxis of leukocytes, phagocytosis, defective T-cell immunity, and fibroblast and epithelial cell dysfunctions [29].

Alcohol (ethanol) intake is another factor that impairs wound healing. Persons who abuse alcohol usage also tend to have very poor eating habits, be malnourished and deficient in most nutrients especially proteins. Consequently, they might exhibit decreased inflammatory and immune responses and type I collagen production, translating to weaker scar tissue during the remodeling phase of wound healing [30]. On the other hand, ethanol intake plays a role in influencing the proliferative phase of wound healing. Angiogenesis of the wound is purported to be the process most impaired by ethanol, along with wound sealing, and collagen production [29]. Although it would seem that, by inhibiting collagen synthesis, high ethanol intake might actually decrease susceptibility to keloids, the reverse is true; by promoting and prolonging wound inflammation, drinking alcohol is more likely to increase susceptibility to keloids [31].

Persons with large body mass index (BMI) due to overweight or obesity are known to have an increased incidence of unfavorable surgical outcomes and complications such as atelectasis, thrombophlebitis, mortality, wound infection, and wound separation [30]. Poor vascularity of adipose tissue is the most plausible explanation for poor wound healing, perhaps associated with decreased tissue oxygen tension [32]. On the other hand, low tissue oxygen tension associated with obesity is likely to upregulate hypoxia-inducible factor-1 (HIF-1) and, together with an exaggerated response to growth hormone and insulin-like growth factor 1 (IGF-1), may contribute to bulky hypertrophic and keloid scarring in susceptible individuals. 

**Figure 4:**
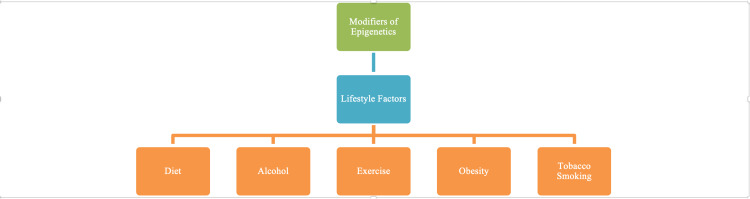
Lifestyle factors impacting wound healing. These factors affect immunity and tissue mediators responsible for wound repair.

One of the foremost effects of tobacco on the skin is vasoconstriction which, like increased adiposity, is likely to decrease tissue oxygen tension and upregulate HIF-1. Nicotine causes vasoconstriction by inhibiting endothelial-dependent, nitric oxide-mediated vasodilation and by reducing tissue perfusion through the stimulation of the thromboxane A2 pathway and the release of catecholamines [33]. Tobacco smoke also increases wound complications by increasing rates of infection. Araco et al. reported in a study of 84 patients that the incidence of infection in smokers was 14.3%, whereas in non-smokers it was only 1.2% [33]. In a meta-analysis, it was determined that the incidence of post-surgical infections and skin necrosis was significantly higher in smokers undergoing breast reduction [33]. Tobacco usage also upregulates reactive oxygen and nitrogen species (RONS) production, associated with premature aging of the skin [34]. Cigarette smoking is known to modify the turnover of the skin’s extracellular matrix resulting in an unevenness of synthesis and degradation of dermal connective proteins such as collagen [34]. Indeed RONS, products of oxidative stress associated with smoking, has been found to downregulate types 1 and 3 collagens, thus upregulating the destruction of collagen [34]. Tobacco also inhibits fibroblast production of transforming growth factor(TGF)β [34], an up-regulator of collagen synthesis, deposition, and bulky hypertrophic or keloid scarring. Figure 4 shows the connection between modifiers of environment-gene (epigenetic) interactions which may result in abnormal wound healing.

Collagen synthesis and how impairments affect wound healing

Fibroblasts and myofibroblasts are cells involved in the healing of wounds in various tissues [35]. During wound healing, fibroblasts in the tissue surrounding the wound are activated and migrate into the provisional matrix containing fibrin and fibronectin [35]. Loss of keratinocytes results in fibroblast activation, synthesis, and release of growth factors such as epidermal growth factor (EGF) and IGF-1 among others during the re-epithelialization phase of wound repair [36].

Collagen is the most abundant protein in human beings, consisting largely of type 1 collagen, which forms fibrillar networks that shape and reinforce tissues such as skin, tendons, and bone [37,38]. Collagen supports the structure and mechanics of tissue and regulates cell proliferation, differentiation, migration, and apoptosis [37]. Fibroblasts are important cells residing in the connective tissues and are responsible for synthesizing collagen in the dermis of the skin or in the lamina propria of mucous membranes, beneath epithelia.

Alterations or insufficient enzymes within the collagen synthesis pathway may predispose individuals to abnormal wound healing that can give rise to hypertrophic scarring and keloids. Collagen is composed of a three-dimensional shape often referred to as a triple helix [39]. The triple helix consists of (Gly-X-Y), with Gly representing the amino acid glycine and “X” and “Y” representing the amino acids proline and lysine [40]. The production of preprocollagen aids in the formation of the triple helix formation. Vitamin C is an essential cofactor for collagen synthesis by facilitating hydroxylation of proline and lysine. A deficiency or inadequate amount of vitamin C can result in scurvy, which is characterized by decreased wound healing in addition to easy bruising and bleeding diathesis [41]. Once proline and lysine residues are hydroxylated, glycosylation occurs and procollagen is then shuttled to the extracellular space by the mechanism of exocytosis. N-and C-terminals are cleaved, thus forming tropocollagen. Lysine side chains are oxidized, leading to the assembly of lysyl-pyridinoline and hydroxylysyl-pyridinoline cross-linking [42]. Collagen synthesis and cross-linking defects are associated with prolonged wound healing and increased collagen synthesis and cross-linking are associated with collagen disorganization and keloids [43].

Effects of disorganized collagen

Wound healing is a complex biological process that restores the skin’s integrity [44]. It is noteworthy that the amount of collagen produced during wound healing differentiates normal scarring from hypertrophic and keloid scarring. Wound healing occurs in three phases: (i) inflammation, (ii) proliferation and (iii) remodeling [45,46]. The first reaction to tissue injury is constriction of blood vessels, leading to activation of fibrin clot formation by platelets; the function of the fibrin clot is to impair blood flow, thus allowing for inflammatory mediators to become activated [46]. Keloid scarring has a strong association with the pro-inflammatory cytokines, IL-1, IL-6, and tumor necrosis factor-alpha (TNF-alpha) [47]. The proliferative phase of wound healing occurs a few days post-injury and the remodeling phase develops three weeks after wound trauma [48].

Disorganized collagen accumulates and hypertrophic scars form when there is an imbalance between ROS and ROS scavengers [49]. Normal wound healing involves early production of parallel fibers of type III collagen followed by a gradual increase in, primarily type I collagen as healing progresses. Hypertrophic scars, however, possess an increased amount of type III collagen usually confined to the borders of the original wound, which contains myofibroblasts, large extracellular collagen filaments, and abundant acidic mucopolysaccharides on histology [45]. Keloid scars possess an extreme increase in wavy, disorganized types I and III collagens which spread beyond the borders of the original wound, and on histology, there are pale-staining hypocellular collagen bundles without nodules or excess myofibroblasts [45]. 

**Figure 5:**
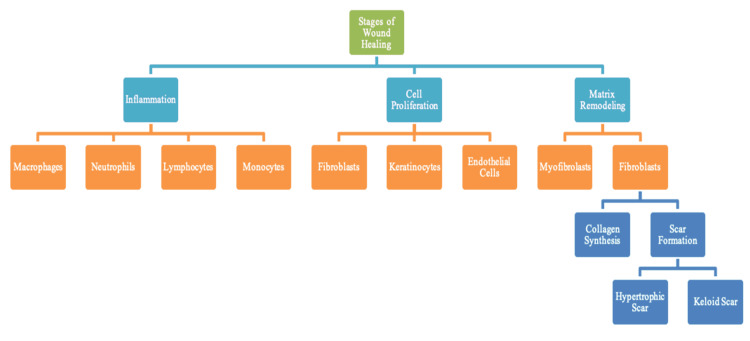
Stages of wound healing regulated by immunologic and biological signals at the initial site of the wound during inflammation. Fibroblasts are found in both cell proliferation and the remodeling phase and are significant for elevated collagen synthesis and scar formation, eventually leading to bulky scar repair.

Regions on the body that do not require as much mechanical stress and tension often do not exhibit keloid scarring. Mechanical force is, therefore, one of the more interesting aspects of keloid scarring. Bulky scarring is commonly seen in areas of the body that require more mechanical stretching, such as the pectoralis major muscles, scapula, shoulder, and suprapubic region of the lower abdomen [7]. The growth pattern of hypertrophic and keloid scarring on a particular area of the body is quite distinct. For example, keloids on the shoulder may have a butterfly distribution, the anterior chest can form a crab’s claw shape and the arm may form the shape of a dumbbell [7]. Keloid scarring also typically affects the upper half of the body. Hypertrophic scarring also develops in areas of high tension, such as the shoulders, neck, pre-sternum, and knees [45]. Hypertrophic scarring often occurs within two months of wound infection or wound closure and has a rapid growth phase for up to six months, with a tendency to regress spontaneously. Keloid scarring, on the other hand, often appears years after minimal trauma and does not regress spontaneously [45]. Figure 5 summarizes the stages of wound healing showing the dell types and critical role of fibroblasts in hypertrophic-keloid scarring.

Ethnicity and specific genetic variants of keloids

Persons of African or Asian ancestry are commonly afflicted with keloid scarring [47]. Recent analyses have been able to identify how certain genetic variants correlate with keloid formation. Individuals affected by keloid scars may inherit keloids through a non-Mendelian, autosomal dominant inheritance involving multiple genes and incomplete penetrance [7]. Various single-nucleotide polymorphisms (SNPs) have been linked to keloid formation. For example, results of a genome-wide association study (GWAS) demonstrate that the keloids in a Japanese family are linked to the 2q23 chromosomal region; whereas, those in an African-American family are linked to 7p11. However, another genome-wide linkage study on a large Chinese family with keloids failed to show the linkage to 7p11; in contrast, linkage intervals at 15q22.31-q23, 18q21.1, and 10q23.31 were found in this family [7]. These two studies demonstrate incomplete penetrance of the disease and that non-mendelian genetic variants may provide more understanding of why certain ethnicities are more prone to keloid scarring.

The role of tissue mediators

Epidermal growth factor receptor (EGFR) stimulates cell growth by using receptor tyrosine kinases for cell proliferation and survival wherein downstream phosphorylation of receptor tyrosine kinases helps to recruit the MAPK and phosphoinositide 3-kinase (PI3K) cell survival intracellular signal transduction pathways [50]. It is noteworthy that EGFR overexpression and dysregulation are linked to certain cancers such as glioblastomas [34] as are some other vital tissue mediators of wound healing. ROS possess the ability to regulate the formation of blood vessels (angiogenesis) at the wound-healing area [23]. Vascular endothelial growth factor (VEGF) is an essential component of angiogenesis. When VEGF concentrations are compared in patients with and without keloid scars, higher VEGF expression in keloids seems to support a hypothesis that VEGF expression plays a role in keloid scarring [51]. 

Wound healing associated with hypertrophic or keloid scars increases the bulkiness of the affected tissues. The bulkiness of scarring is attributed primarily to the dysregulation of collagen deposition in the extracellular matrix. Increased collagen synthesis is shown to result, primarily, from prolonged inflammation. Keloids, histologically, are characterized by increased twists of condensed, hyalinized bundles of collagen. In keloids, collagen synthesis is about 20 times greater than in healthy skin. TGFβ and platelet-derived growth factor (PDGF) are key players in abnormal scar responses such as that in the case of keloid formation [52]. There are various signaling pathways that have been identified as playing major roles in inflammation and increased collagen synthesis. The signal transducer and activator of transcription(STAT)-3 signaling pathway consists of a variety of cytokines and is involved in cell proliferation, differentiation, apoptosis, migration, fibrosis, and inflammation. In hypertrophic scars, the STAT-3 pathway was seen to upregulate procollagen COLIA2 [53]. Endothelial dysfunction may also play a role in surges of inflammatory cells, leading to the overproduction of collagen, resulting in abnormal scar pathogenesis [7]. In that regard, it is noteworthy that keloids seem to occur more often in patients exhibiting clinical endothelial dysfunction [54]. The finding that Lumican, a collagen fibril assembly regulator, and collagen V levels appear to be highly overexpressed in keloid tissues [55] support the hypothesis that increased collagen levels contribute to keloid and hypertrophic scarring.

ROS act in the host’s defense via phagocytes which induce a ROS-mediated oxidative burst leading to the destruction of pathogens and leakage of excess ROS into the surrounding area with bacteriostatic effects [23]. The fibroblast proliferation and respiratory burst associated with keloids result in an imbalance between oxygen delivery and utilization, thereby producing a condition of hypoxia, oxidative stress, and increased production of ROS. Adaptation to hypoxic conditions is partly mediated by the production of hypoxia-inducible factor-1 (HIF-1) [54]. Under hypoxic conditions, HIF-1 acts as a transcription factor and aids cell survival by inducing the expression of genes that produce the aforementioned chemical mediators that contribute to cell proliferation, metabolism, and angiogenesis [56].

**Figure 6:**
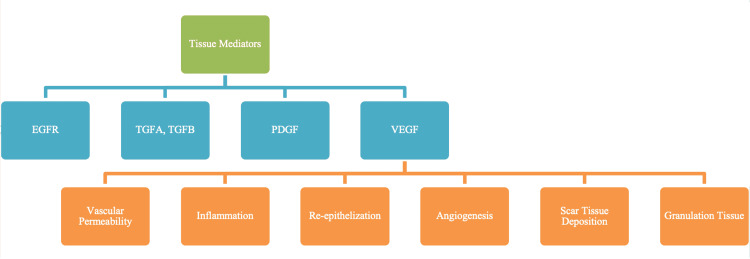
Tissue mediators contributing to abnormal wound healing and hypertrophic-keloid scarring. VEGF is the prime tissue mediator that assists with scar tissue deposition; elevated VEGF expression is associated with a predilection for keloid scarring. EGFR: Epidermal growth factor receptor; TGFA: Transforming growth factor-alpha; TGFB: Transforming growth factor-beta; PDGF: Platelet-derived growth factor; VEGF: Vascular endothelial growth factor

TGFβ induces angiogenesis, helps to generate wound fibrosis [4,5], and appears to influence the dysregulation of collagen remodeling in the scar healing process [57], a characteristic of keloids. TGFα, TGFβ, and PDGF are key cytokines in cell chemotaxis and angiogenesis, as well as mitogenicity of keratinocytes and fibroblasts. PDGF also stimulates wound contraction and TGFβ also stimulates fibroblasts. There are three types of TGFβ; specifically, TGFβ type1 has been linked to an increased level of collagen and fibronectin synthesis by fibroblasts [58]. When overexpressed, TGFβ type1 also stimulates CTGF activity. CTGF binds directly to integrins, the principal proteins by which cells bind to the extracellular matrix (ECM) and is defined as an ECM-associated heparin-binding protein. Synthesized by fibroblasts, this growth factor stimulates chemotaxis and cell proliferation and has been associated with the formation and development of hypertrophic and keloid scars when there is increased expression of CTGF [6]. PDGF, EGF, and fibroblast growth factor (FGF) combine to induce DNA synthesis within keloid fibroblasts [59]. The formation of keloids would occur at trauma sites or regions of the skin where there is tension or motion. However, keloids have also been reported to form in regions where there is no obvious trauma, thereby implying a role for the release of PDGF in such keloids [59]. Figure 6 summarizes the main tissue mediators shown to be involved in abnormal wound healing and hypertrophic-keloid scarring. 

Near-infrared fluorescence spectroscopy detects keloids diagnostically

Near-infrared fluorescence spectroscopy (NIRS) is shown to have the potential to detect keloids [60]. Based on findings that RONS contribute to keloid formation, two cyanine-based dyes, one conjugated to boronic acid (CyBA) and the other to trifluoromethyl (CyTF) moieties, appear to be capable of differentiating normal dermal fibroblasts from keloid-producing fibroblasts [60]. In the presence of peroxynitrite, an oxidizing agent and biochemical marker for oxidative stress, the CyTF probe exhibits specificity for peroxynitrite (ONOO^-^) compared to CyBA, which is less specific and responsive to the presence of hydrogen peroxide and/or peroxynitrite [60]. CyTF produces a greater-than-two-fold increase in the NIRS signal in keloid fibroblasts. A proof-of-concept experiment demonstrates markedly decreased CyTF-NIRS signaling after treatment with the TGFβ receptor-1 inhibitor RepSox, a molecule that can replace the sex-determining region Y (SRY) box 2 (Sox2) transcription factor for the maintenance and reprogramming of pluripotent stem cells [60]. This experiment clearly shows the ability of NIRS to detect physiological modulation of keloid fibroblast activation; thereby suggesting that this technological advancement is likely to have the potential to improve the diagnosis and treatment of keloid scarring.

Significance of skin types in hypertrophic and keloid scarring

Melanogenesis is the process by which the pigment melanin is produced, melanin is a phenotypic trait that differentiates the colors of hair, skin, and eyes [61]. Melanin is made within the skin’s melanocytes in organelles known as melanosomes, which function to protect the cell from oxidative stress during synthesis [62]. Melanocytes are derived from neural crest cells after the closure of the neural tube during embryological development [2,3]. Melanocytes are found in the skin, iris, pigmented layer of the retina in the eye, cochlea of the inner ear, and meninges, and they share a common lineage with Schwann cell precursors and neurons. It is noteworthy that neuromelanin-pigmented cells are found in the brain parenchyma’s catecholaminergic cells of the substantia nigra pars compacta and locus coeruleus, where it is thought to act as a chelator and neuroprotectant against heavy metals and oxidative stress. This neural crest-epidermis-brain connection likely accounts for the high rate of cutaneous melanomas metastasizing to the central nervous system.

Skin pigmentation is determined by the differences in the number, size, and shape of melanosomes, although all humans have about the same number of melanocytes [63]. There are two types of melanin in the skin: pheomelanin and eumelanin [64]. Pheomelanin is a yellow-red pigment; whereas, eumelanin contains a brown-black pigment [65]. The Fitzpatrick system is widely used to categorize skin types. Fitzpatrick skin types IV-VI are common in dark-skinned persons who are most susceptible to dyschromia, post-inflammatory hyperpigmentation (PIH), melasma, and keloids [2,3]. Additionally, persons of Asian descent are more prone to freckles, melasma, and PIH [63]. Keloid scarring has an incidence of 6%-16% in African populations [45]. The mechanism of bulky scar repair having a predilection for Fitzpatrick skin types IV-VI is not well understood, however, further research may assist with better treatment outcomes for individuals afflicted with this condition.

Exercise, epigenetics, and tissue repair

Exercise is known to increase the production of RONS and various pro-inflammatory cytokines. Mechanisms reported as contributory to this exercise-induced production of RONS include insufficient electron transfer through the mitochondrial respiratory chain, auto-oxidation of heme proteins, processes leading to inflammation, and conditions leading to ischemia-reperfusion injury and activation of xanthine oxidase [1]. RONS also play major roles in activating nuclear factor erythroid-2 related factor-2 (Nrf2), a transcription factor that promotes the production of heme oxygenases and a number of intracellular antioxidants such as glutathione. Nrf2 is regulated by Kelch-like ECH-associated protein 1 (Keap1), it promotes Nrf2’s ubiquitination and proteasomal degradation [66]. Keap1 is a cysteine-rich protein modified by electrophiles and oxidants. This sensitivity causes conformational changes to Keap1, therefore, stabilizing the Keap1-Nrf2 interaction. These conformational changes prevent proteasomal degradation which in turn increases Nrf2 accumulation in the nucleus where it binds with antioxidant response elements (AREs). This binding regulates the transcription of over 250 genes involved in the antioxidant response, inflammation, and cell differentiation [66]. Nrf2 plays a significant role in wound healing.

Transcription factors Nrf2 and NF-κB (nuclear factor kappa-light-chain-enhancer of activated B cells) appear to be instrumental in repair-associated inflammation control following injury [67]. Many studies have been carried out aiming to assess Nrf2’s function in wound healing. Nrf2 gain and loss of function have various effects on wound healing. When there is a loss of Nrf2 function, it has been observed that there is a delayed induction of cytokines, prolonged wound inflammation, and reduced collagen deposition on a global and keratinocyte level. However, when there is a gain of Nrf2 function, it has been observed that there is increased keratinocyte proliferation, restoration of normal TGFβ1 levels, accelerated wound closure, expansion of pilosebaceous cells, earlier onset of fibroblast senescence, as well as increased keratinocyte proliferation [68]. These findings suggest that Nrf2 is likely to play an essential role in mediating wound closure and healing. In response to DNA damage in lymphocytes, it is reported that fewer DNA strand breaks found in the trained athletes could be attributed to RONS stimulation of Nrf2. Exercise is found to induce more DNA strand breaks and DNA strand breaks are repaired faster in the lymphocytes of untrained persons than in those of trained athletes [69]. Extrapolating this finding to wound healing, it seems logical that exercise-induced production of RONS and Nrf2 is likely to have a beneficial effect by limiting inflammation and deposition of collagen in individuals who are known to have predilections for bulky hypertrophic and keloid scarring. ROS has been observed to play crucial roles in the process of wound healing responses by acting as messengers for immunocytes and non-lymphoid cells which play roles in the repair process by directing lymphoid cell recruitment to the site of the wound. In tissue repair, ROS are thought to mediate cell division and the migration of keratinocytes and endothelial cells, along with the formation of collagen [23]. In that regard, gelatin (collagen hydrolysate) protein combined with vitamin C supplementation is reported to increase collagen synthesis in healthy non-sedentary, exercising adult males [70]. Increased amounts of exercise and slow-wave (reparative phase) sleep increase growth hormone secretion from the anterior pituitary [71-73] and growth hormone is also a potent stimulator of collagen synthesis. Several studies have shown that growth hormone stimulates the synthesis of collagen type I and II; for example, recombinant human growth hormone (rhGH) administered to healthy young individuals over a 14-day period increased serum growth hormone, serum IGF-1, IGF-1 mRNA expression in skeletal muscles and tendons [71]. rhGH administered to healthy elderly men to determine the effects of growth hormone on tendon collagen synthesis in the elderly used two injections of rhGH into patellar tendons, and 6-hours post-injection it was observed that the tendon collagen fractional synthesis rate was increased in 10 of the 12 study subjects [74]. However, whether gelatin, vitamin C, or growth hormone supplementation, with or without exercise, has the capacity to increase collagen deposition and contribute to bulky scarring remains unknown. Growth hormone’s effects are mediated by IGF-1, the synthesis of which is shown to be upregulated in both hypertension and overweight/obesity. IGF-1 functions as a proliferative and differentiation factor (oftentimes via the MAPK or PI3K signaling pathway) affecting cell survival, protein synthesis, and energy utilization [75]. Hypertension is involved in two phases of pathological wound healing: the proliferation and remodeling phases. In wounded hypertensive rats, a massive and instant increase in blood flow was observed compared to normotensive rats, associated with capillary proliferation [76]. The link between hypertension and keloid severity has been studied in a cohort of 304 hypertensive patients with keloids wherein blood pressure was found to be significantly linked with keloid number and size [77]. Obesity is reported to have effects on the structure and function of collagen, thereby affecting wound healing. In that regard, obese rats appear to have weaker skin than normal-weight rats; i.e., a less tensile force due to the failure of collagen accumulation at the wound site [78]. There are also serious circulation changes linked to obesity that may lead to the increased incidence of keloid formation wherein slowing of blood flow to a wound site impairs wound healing, thereby increasing susceptibility to keloids [79], possibly by an ischemic mechanism. 

Importance of perioperative dietary modification

Dietary modifications, perioperatively, are thought to be beneficial in patients undergoing surgery. It’s no mystery that diet plays a major role in all things, wound healing included. Ensuring that one obtains all macro-and micro-nutrients from their diet is imperative in wound healing. Vitamins such as A, B1, B2, C, E and minerals such as iron, copper, zinc, magnesium, manganese, and silicon all play roles associated with the activity of collagen in the healing process. Such nutrients serve as cofactors in collagen cross-linking, collagen synthesis/formation, and contribute to procollagen synthesis [80,81]. Macronutrients (carbohydrates, fats, and proteins), especially proteins, are needed to stimulate immune system cells such as macrophages, phagocytes, leukocytes, monocytes, and lymphocytes, which require structural and functional proteins (e.g., enzymes) to be metabolically active. Dietary protein deficiency decreases fibroblast production and collagen synthesis for wound healing. The amino acid arginine stimulates several processes such as cell growth, protein synthesis, and collagen deposition require arginine. Glutamine is among the most prevalent amino acids in plasma, playing an important role in the production of nucleotides in fibroblasts, macrophages, and epithelial cells. During the inflammatory phase of wound healing, glutamine also stimulates inflammatory responses [81]. Severe undernutrition extends the inflammatory phase that may increase the probability of keloid scarring by the mechanism of increased synthesis of collagen [82]. Energy (caloric intake) plays an important role in wound healing as well; glucose is the major source of fuel for the synthesis of collagen [81].

Stem cells and keloids

A model of keloid formation proposes that the cytokine TGFβ creates conditions for overproduction of extracellular matrix by the mechanism of endothelial-mesenchymal transition, regulated by an injured tissue’s renin-angiotensin system (RAS) [83]. Evidence is emerging that virtually all tissues possess a local RAS, probably needed for fine-tuning the particular tissue’s sodium-potassium balance. Angiotensin II is the “workhorse” of the RAS by virtue of its multifunctional effects on aldosterone and sodium-potassium balance, thirst and water balance, cardiac hypertrophy and fibrosis, etc. [84]. Angiotensin’s actions on cardiac fibroblasts make it a significant contributor to the heart remodeling associated with virtually every cardiovascular disease. It is, therefore, quite plausible that keloid and other hypertrophic scarring is associated with upregulation of the local tissue RAS. Indeed, an angiotensin-converting enzyme inhibitor used for treating hypertension is reported to improve keloid scarring [85]. TNFα, an important effector of the innate immune system, appears to be upregulated in keloid tissues and also induces fibroblast production of TGFβ [86]. These findings provide a foundation for novel hypotheses and a unified hypothesis concerning the roles of stem cell, endocrine and immunologic signaling in bulky scarring to fuel future research.

## Conclusions

Interactions between several biochemical and immunological signaling pathways for increased collagen synthesis and hypertrophic-keloid scarring are associated with the matrix remodeling stage of wound healing. Hypertrophic-keloid scarring is a complex phenomenon resulting from epigenetic processes involving histones, DNA methylation, lifestyle modifiers of DNA methylation, oxidative stress mediated by ROS, and various tissue mediators such as TGFA, TGFB, PDGF, and VEGF. Clinicians should consider the multifarious environment-gene interactions which may contribute to the development of hypertrophic-keloid scarring when counseling patients about the healing of wounds associated with accidental, surgical, and tattooing injuries to the skin.
